# Immunoarchitectural patterns as potential prognostic factors for invasive ductal breast cancer

**DOI:** 10.1038/s41523-022-00389-y

**Published:** 2022-02-28

**Authors:** Xue Du, Zhe Zhou, Yun Shao, Kun Qian, Yongfang Wu, Jun Zhang, Miao Cui, Jingjing Wang, Shengqi Wang, Yanhong Tai

**Affiliations:** 1 Beijing Institute of Radiation Medicine, Beijing, PR China; 2 Department of Pathology, Fifth Medical Center of Chinese PLA General Hospital, Beijing, PR China

**Keywords:** Breast cancer, Prognostic markers

## Abstract

Currently, tumor-infiltrating lymphocytes (TILs) in invasive breast cancers are assessed solely on the basis of their number, whereas their spatial distribution is rarely investigated. Therefore, we evaluated TILs in 579 patients with invasive breast cancer of no special type (IBC-NST) with a focus on their spatial distributions in tumor center (TC) and invasive margin (IM). We also assessed a new factor, namely para-tumor infiltrating lymphocytes (PILs) in the para-tumor lobular area (Para). Five immunoarchitectural patterns (IPs) were observed, which were significantly associated with clinicopathological features, especially molecular subtypes, histological grades, clinical stages, and programmed death-ligand 1 (PD-L1) expression. High-TIL density (IP1/2) correlated with favorable disease-free survival (DFS) in TNBC patients (*p* = 0.04), but opposite results were observed for luminal B subtype patients (both the lowest TIL and PIL densities (IP5) correlated with good DFS, *p* = 0.013). Luminal B patients with high TILs in the IM and low TILs in the TC (IP3) exhibited the worst DFS, whereas those with low TILs (similar to IP5) and high PILs (IP4) exhibited poor DFS. We also identified TIL subpopulations with significantly different IPs. Our findings suggest that IP can be a potential prognostic factor for tumor immunity in IBC.

## Introduction

Breast cancers are clinically and molecularly heterogenous, with 5–10 intrinsic subtypes^1^. Each subtype displays varied molecular characteristics that form the basis for therapeutic resistance^2,3^ and different therapeutic strategies^4^. Immunotherapy and combined neoadjuvant chemotherapy are being aggressively developed, with anti- programmed death-ligand 1 (PD-L1) exhibiting strong immunomodulatory therapeutic potential against breast cancer^5^. A pre-existing immunological response might enhance the efficacy of conventional cytotoxic chemotherapy^5–7^. However, despite accumulating evidence, the translation from basic tumor immunology to clinical practice remains problematic^7^. PD-L1 and tumor mutational burden (TMB)-based immunotherapeutic clinical trials have shown favorable results in a small subset of invasive breast cancer (IBC) patients, mainly triple-negative breast cancer (TNBC) patients^8^. Previous studies have shown that high count of tumor-infiltrating lymphocytes (TILs) cannot constantly warrant a good outcome in all IBC patients. In luminal-HER2-negative patients, high TIL count is considered an adverse prognostic factor for survival^5^; however, the TILs should be studied with a new perspective for a comprehensive understanding of the tumor microenvironment.

A recent investigation into more reliable predictors^9^ revealed that immune contextures, such as TIL density and spatial localization, are associated with clinicopathological characters and PD-L1 expression based on molecular subtypes, and were therefore considered appropriate immunotherapeutic candidates. However, the association of clinicopathological characters with para-tumor infiltrating lymphocytes (PILs) located in the para-tumor lobular area (Para) remains uncertain. Therefore, the quantitative molecular and spatio-morphological parameters of infiltrating lymphocytes interactions should be explored to improve the identification of predictive markers.

In this study, we investigated hematoxylin and eosin (H&E)-stained sections of 579 tissue samples from invasive breast cancer of no special type (IBC-NST) patients to define tumor immunoarchitectural patterns (IPs) and TIL density. Therefore, comprehensive analysis of the identified IPs was performed with respect to lymphocyte density, location, immunophenotyping, and combined histopathological characteristics (such as the histological grade, clinical stage, molecular type, and survival status) in patients with IBC-NST.

## Results

### Stratification of IBC-NSTs into five IPs

**Fig. 1:**
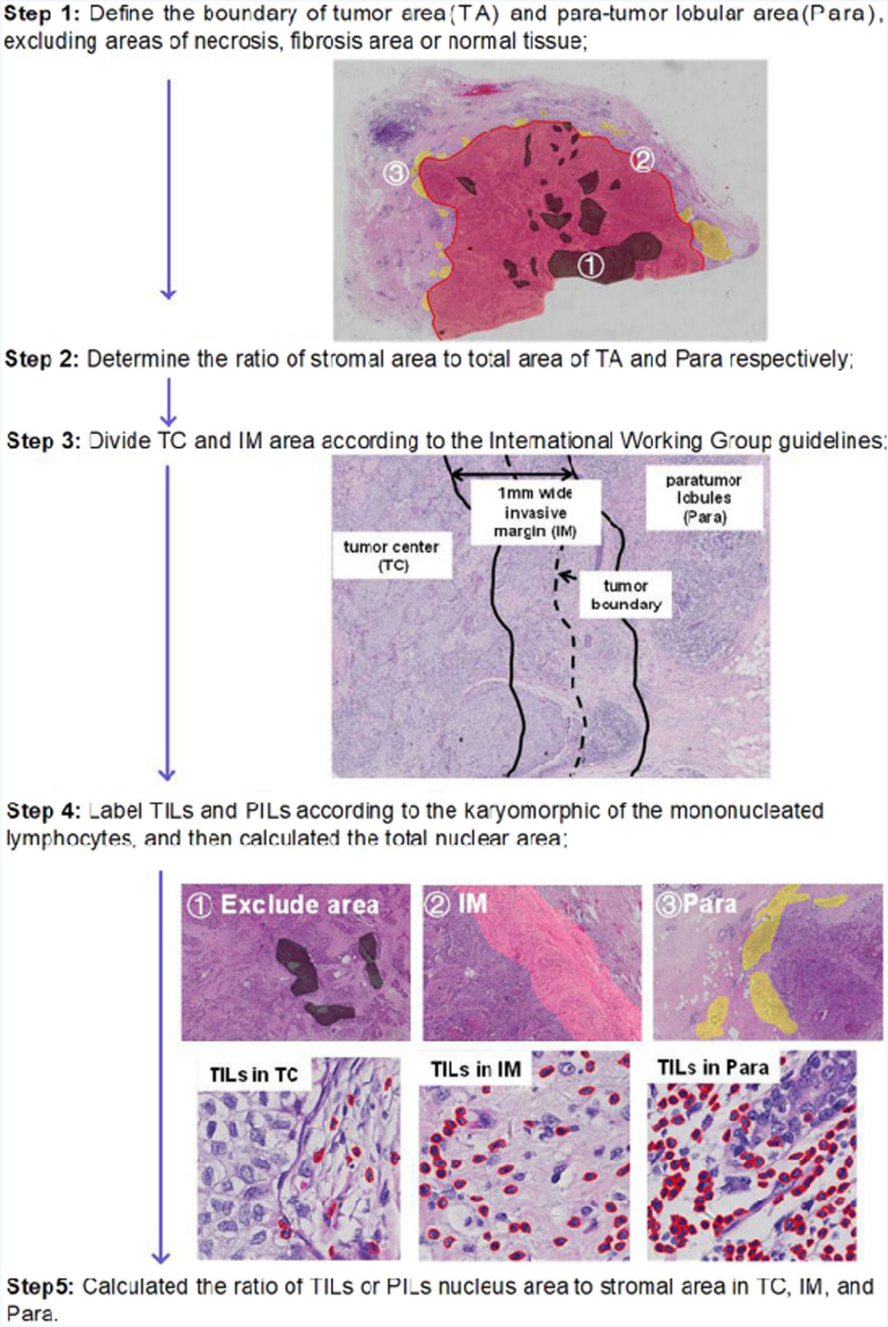
Outline of the traditional software-assisted assessment procedure used for analyzing TILs and PILs in IBC-NST samples. TILs tumor-infiltrating lymphocytes, PILs para-tumor infiltrating lymphocytes, IBC-NST invasive breast cancer of no special type.

**Fig. 2:**
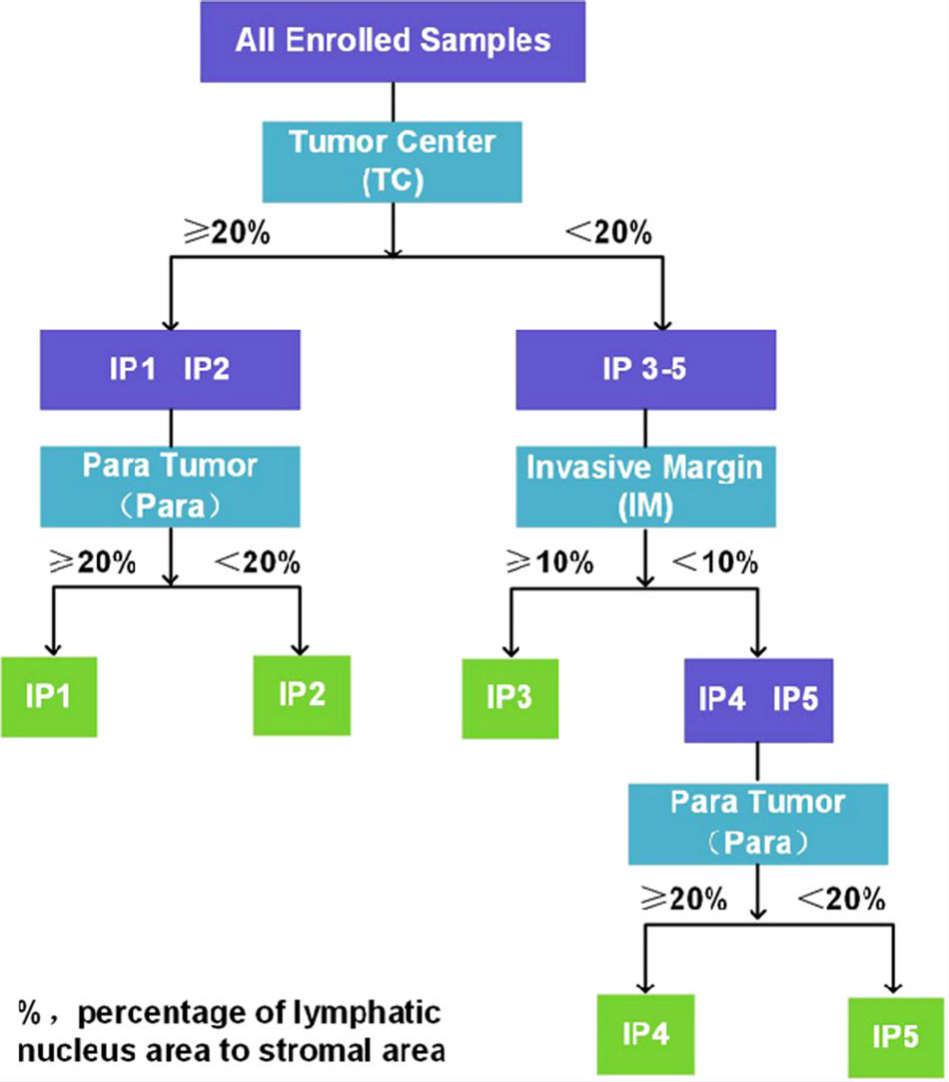
Flow chart of the process used to identify five different immunoarchitectural patterns. IP immunoarchitectural pattern.

**Fig. 3:**
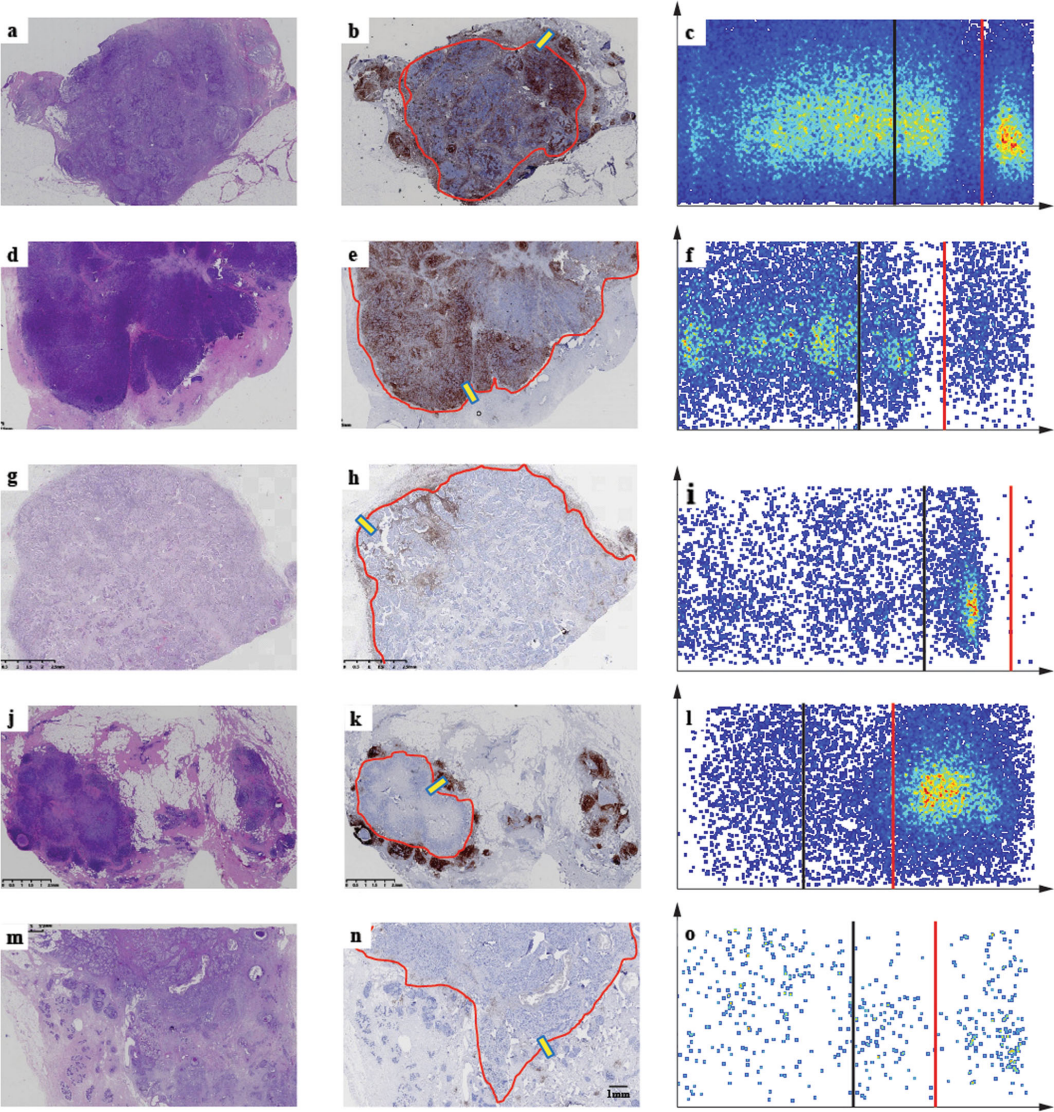
Schematic diagram of immune cells’ distribution and morphology in five IP patterns of breast cancer tissues. H&E-stained sections, IHC sections, and TissueGnostics analysis of IP1 (**a**–**c**), IP2 (**d**–**f**), IP3 (**g**–**i**), IP4 (**j**–**l**), and IP5 (**m**–**o**). The IHC sections were stained for the LCA marker. The red lines in the IHC sections show the margins of the tumor areas. TissueGnostics analysis of the yellow frames in panels **b**, **e**, **h**, **k**, and **n** is shown in **c**, **f**, **i**, **l**, and **o**, respectively. The black and red lines shown for the TissueGnostics analysis represent the margins of the tumor areas and para-tumors, respectively. H&E hematoxylin and eosin, IHC immunohistochemistry, IP immune pattern, LCA leukocyte common antigen.

We assessed 579 primary IBC-NST cases for tumor immunity and grouped them into five IPs, as indicated in the flowchart (Figs. 1 and 2). IP2 (19/579, 3.28%) had the least number of cases, followed by IP1 (69/579, 11.92%), IP3 (110/579, 19.00%), IP4 (130/579, 22.45%), and IP5 (251/579, 43.35%) (Table 1). We displayed the cross-referenced H&E and leukocyte common antigen (LCA) stained sections, and TissueGnostics images of typical cases to highlight the distinct differences of five IPs in Fig. 3.

**Table 1:** Population clinicopathological characters.

Characteristics		No.	Percentage	Characteristics		No.	Percentage
Age	≤35	32	5.53%	TNM	I	269	46.46%
	35–50	218	37.65%		II	227	39.21%
	≥50	329	56.82%		III	74	12.78%
					IV	7	1.21%
Sex	Female	578	99.83%		Unknown	2	0.35%
	Male	1	0.17%				
				ER status	Negative	118	20.38%
Postmenopausal	not yet	312	53.89%		Positive	461	79.62%
	Postmenopausal	260	44.91%				
	Unknown	6	1.04%	PR status	Negative	170	29.36%
	Null (male)	1	0.17%		Positive	409	70.64%
Family genetic history	not	560	96.72%	HER2 status	Negative	474	81.87%
	Family genetic history	15	2.59%		Positive	105	18.13%
	Unknown	4	0.69%				
				Ki67	≤14%	151	26.08%
Tumor size	T1( ≤ 2)	379	65.46%		å 14%	428	73.92%
	T2(2–5)	155	26.77%				
	T3( ≥ 5)	10	1.73%	Molecular type	Luminal A	136	22.70%
	T4	33	5.70%		Luminal B	272	45.41%
	Unknown	2	0.35%		Luminal HER2	58	9.68%
					HER2+	47	7.85%
Lymho node	N0	377	65.11%		TNBC	66	11.02%
	N1	143	24.70%				
	N2	38	6.56%	Immuno- architectural Pattern	1	69	11.92%
	N3	21	3.63%		2	19	3.28%
					3	110	19.00%
Metastasis	M0	572	98.79%		4	130	22.45%
	M1	7	1.21%		5	251	43.35%

### Lobular involvement, cancerous embolus, and histological grade

**Fig. 4:**
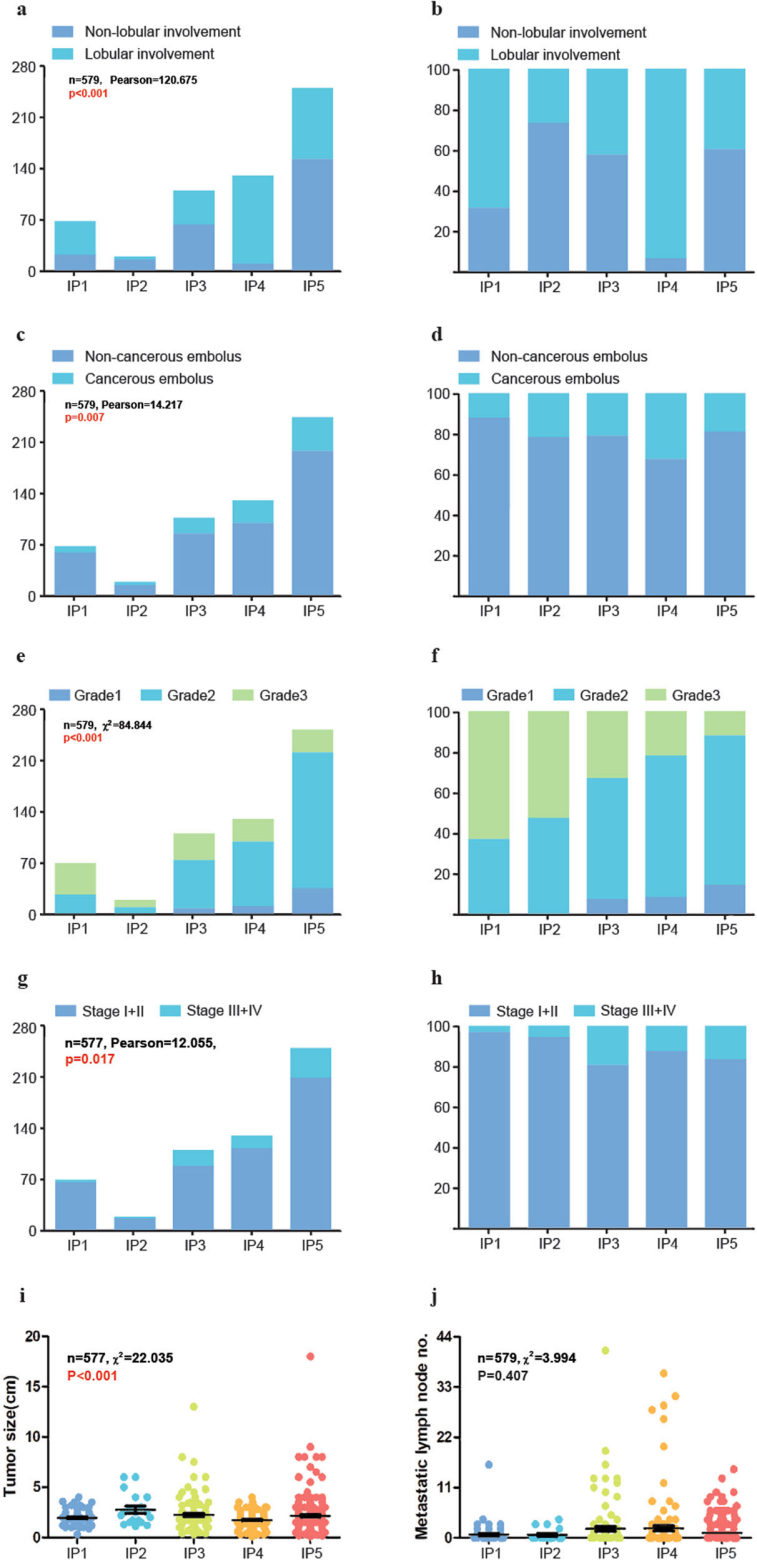
Major clinical characteristics analysis in five immunoarchitectural patterns. Lobular involvement, cancerous embolus, tumor histological grade, clinical stage, tumor size, and number of metastatic lymph nodes in five immunoarchitectural patterns, in terms of distributions (**a**, **c**, **e**, **g**, **i**, **j**) and percentages (**b**, **d**, **f**, **h**). The correlations between patterns and categorical variables (including tumor grades, molecular subtypes, lobule cancerization, and vascular invasion) were analyzed by the χ² test (for trends) or Fisher’s exact test. Continuous variables (tumor size and number of metastatic lymph nodes) were assessed by the Kruskal–Wallis H tests under K independent-sample tests.

IP4 had significantly higher frequency of lobular involvement (121/130, 93.08%) compared with that in the other four IPs. A similar trend was also observed with IP1 (47/69, 68.12%), which had high PIL counts similar to IP4, but unlike IP2 (5/19, 26.32%), IP3 (46/110, 41.82%), and IP5 (98/251, 39.04%) (Fig. 4a, b, Supplementary Table 1). Moreover, the cancerous embolus ratio was also significantly higher in IP4 (42/130, 32.31%) than that in IP1 (8/69, 11.59%), IP3 (23/110, 20.91%), and IP5 (47/251, 18.73%) (Fig. 4c, d, Supplementary Figure 2, Supplementary Table 1).

The histological-grade distribution differed significantly between each of the five IPs (χ² = 84.84, *p* < 0.001; Fig. 4e, f). No grade 1 cases were found in the IP1 and IP2 groups, and grade 3 cases (62.32% in IP1 and 52.63% in IP2) were more common than grade 2 cases (37.68% in IP1 and 47.37% in IP2). In contrast, in IP5, grade 1 (14.34%) and grade 2 (73.71%) cases accounted for 88.05% of all cases, whereas grade 3 cases accounted for only 11.95% of the cases (Supplementary Table 1). The grade distribution was not different between IP3 and IP4, both of which exhibited more grade 3 cases and fewer grade 1 cases compared to IP5. No significant differences were identified between the IP1 and IP2, and IP2 and IP3 pairs (Supplementary Fig. 2).

### Clinical stage, tumor size, and lymph node metastasis

Tumor-size measurements were missing in one case each for IP4 and IP5; thus, these cases were omitted from subsequent analyses. Most patients (496) exhibited early stage (stage I, 269 cases or stage II, 227 cases) disease, and only 81 patients exhibited late-stage disease (stage III, 74 cases or stage IV, 7 cases). A significant difference was found between patients exhibiting early- and late-stage disease among the 5 IPs (χ² = 12.055, *p* = 0.017). The proportion of late-stage cases in IP1 (2.90%) was significantly lower than that in IP3 (19.09%), IP4 (12.40%), and IP5 (16.40%) (Fig. 4g, h, Supplementary Table 1). Only one late-stage case was found in IP2 (1/19, 5.26%), although it was not significantly different from other IPs (Supplementary Fig. 2).

Tumor sizes differed significantly between the 5 IPs (χ^2^ = 22.035, *p* < 0.001), with the lowest median tumor size in IP4 and the highest in IP2 (Fig. 4i, Supplementary Fig. 2, Supplementary Table 1). The differences were analyzed in terms of the number of lymph node metastases, but no statistical significance was found (χ^2^ = 3.994, *p* = 0.407. Fig. 4j, Supplementary Fig. 2).

### Molecular subtype

**Fig. 5:**
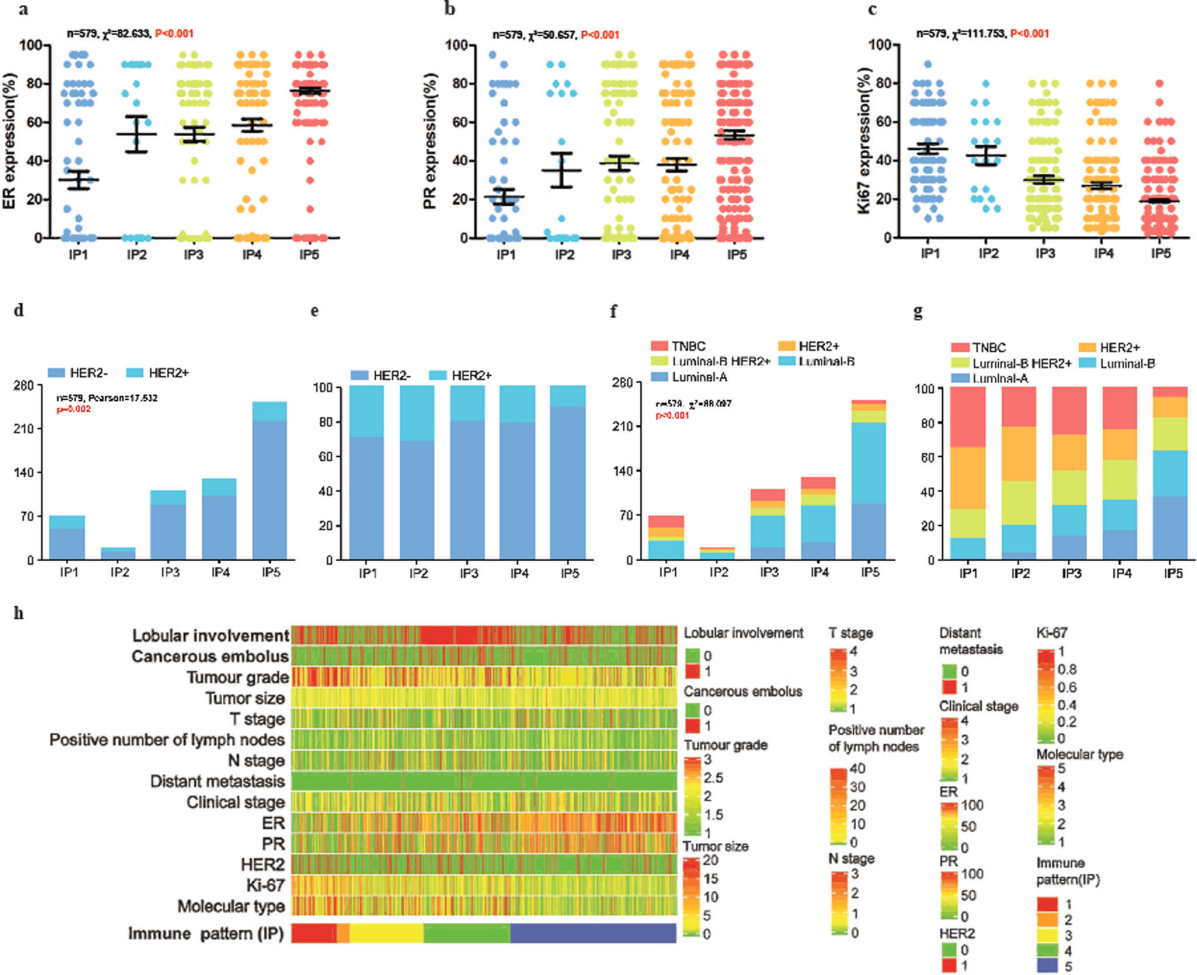
ER, PR, Ki-67 expression and HER2 status in five immunoarchitectural patterns and heatmap of all enrolled patients’ clinical characteristics. ER (**a**), PR (**b**), Ki-67 (**c**) expression and HER2 (**d**) status in five immunoarchitectural patterns The percentages of HER2-positive cases for each pattern (**e**). Distributions (**f**) and percentages (**g**) of molecular subtypes in each of the five IPs. **h** Heatmap of the clinical characteristics of all 579 patients included in this study. Molecular type, 1, luminal A subtype; 2, luminal B subtype; 3 represent luminal B HER2 subtype; 4 represent HER2 subtype; and 5 represent TNBC subtype.

Immunohistochemistry (IHC)-staining of samples from 579 patients revealed that the ER- and PR-expression levels gradually increased from IP1 to IP5 (Fig. 5a, b, Supplementary Fig. 2), whereas the proliferation indicator Ki-67 revealed an opposite trend (Fig. 5c). HER2 + cases were significantly lower in IP5 than in the other IPs (Fig. 5d, e).

Among the 136 luminal A subtype samples, most were distributed in IP5 (88/136, 64.71%), followed by IP4 (27/136, 19.85%), and IP3 (19/136, 13.97%) (Supplementary Table 1). Only one case each of luminal A subtype was found in IP1 and IP2. Conversely, patients with the TNBC or HER2 + subtype accounted for a greater proportion of IP1 (19/69, 27.54% and 14/69, 20.29%, respectively) compared to that of IP2 (each 3/19, 15.79%), IP3 (19/110, 17.27% and 10/110, 9.09%, respectively), IP4 (19/130, 14.62% and 10/130, 7.69%, respectively), and particularly IP5 (6/251, 2.39% and 10/251, 3.98%, respectively). Luminal B subtype and luminal-HER2 subtype cases were distributed evenly into the five IPs (Supplementary Table 1). Pearson’s chi-square test of molecular subtypes revealed significant differences between IPs (χ² = 88.097, *p* < 0.001; Fig. 5f, g), and IP5 contained significantly more mild subtype cases than other IPs, whereas aggressive subtype cases were significantly more common in IP1 than IP3–IP5. The IP2 group showed no significant difference in the molecular-subtype distribution with other IPs, except for IP5 (Supplementary Fig. 2). The distribution of IPs in the luminal B subtype differs significantly in three histological grades (χ^2^ = 18.494, *p* = 0.018).

### Pattern integration

**Fig. 6:**
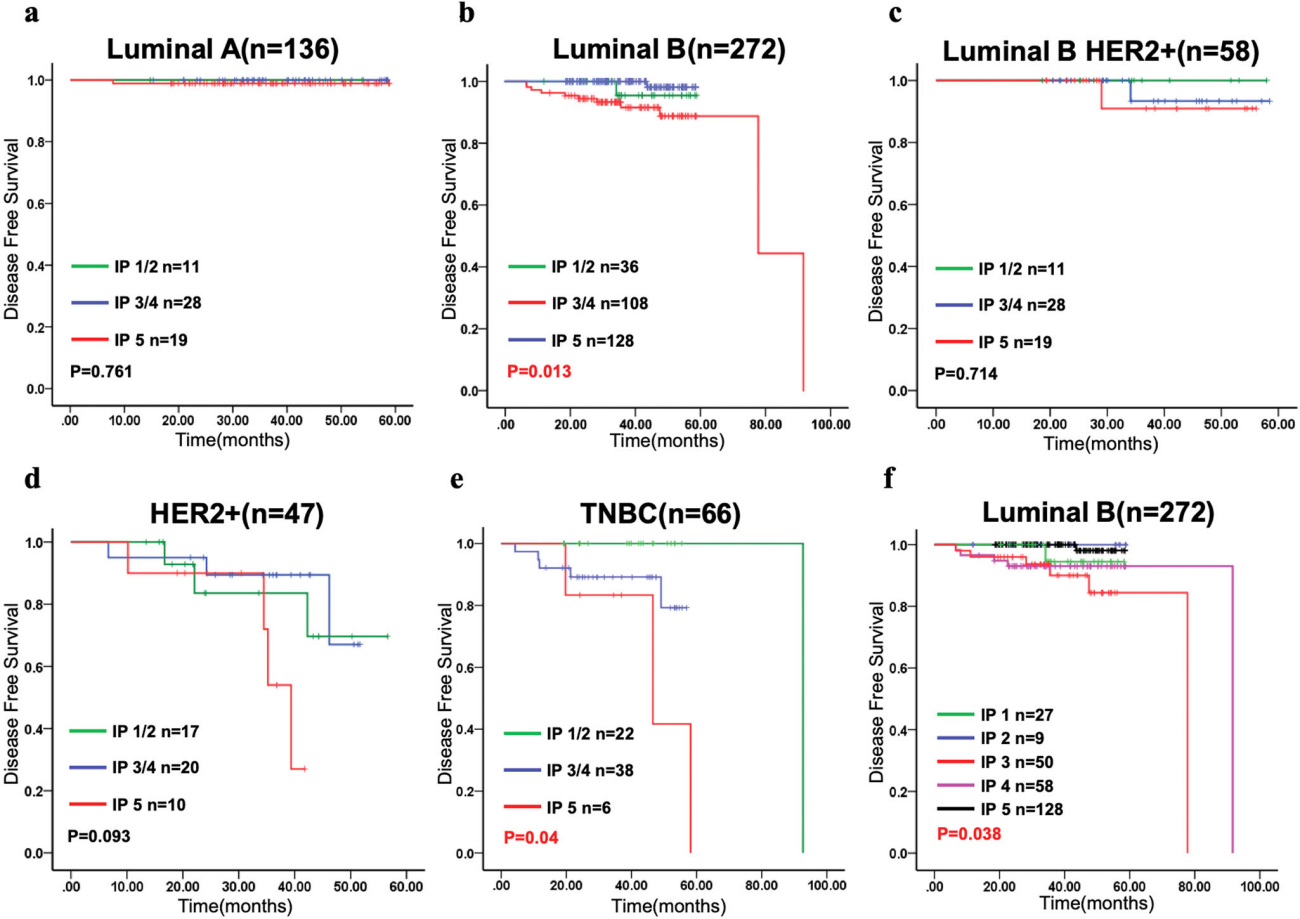
Kaplan–Meier curves for disease-free survival for all patients according to the molecular types. **a**–**e** Log-rank (Mantel–Cox) χ² test = 3.054, *p* = 0.217; Breslow (generalized Wilcoxon) χ² square test = 8.724, *p* = 0.013; Tarone–Ware χ² test = 8.724 *P* = 0.049. Kaplan–Meier curves for luminal B subtype in all five patterns **f**.

The clinicopathological characteristics of the 579 patients were summarized by heatmapping (Fig. 5h). Although the IP2 cases shared similar prognosis-related clinicopathological characteristics with the IP1 cases (such as a higher histological grade, more aggressive molecular subtype, and lower clinical stage), the differences between IP2 and IP3–5 were less significant than those between IP1 and IP3–5 (Supplementary Figure 2). As there were only 19 IP2 cases (and even less when subdivided), the low degree of significance may partially reflect the small sample size. We found that the curve for IP5 was unique, whereas the curve tendencies of IP1 and IP2, as well as IP3 and IP4, were highly similar (Supplementary Figure 3). These three characteristics are the major factors that affect disease-free survival (DFS); thus, we merged the IPs into three groups (IP1/2, IP3/4, and IP5) as prognostic groups for subsequent DFS analysis (Fig. 6).

### DFS analysis

In this study, Formalin-fixed, paraffin-embedded (FFPE) tissues were collected within 3 years to meet the quality requirements for nucleic acid-isolation for genome-wide mRNA-expression analysis and exome sequencing. Given the short duration of the disease, we only assessed the IPs as a prognostic factor for DFS, with a median survival time of 91.63 months (IQR 69.40–113.86). No significant association was found with DFS among the five IPs. When stratified by molecular subtypes, the three merged-IP groups exhibited significant associations with DFS (log-rank = 3.054, *p* = 0.217; Breslow = 8.724, *p* = 0.013; Fig. 6a–e). In the luminal B subtype, all three merged IPs (log-rank = 8.711, *p* = 0.013; Fig. 6b) and all five individual IPs (log-rank = 10.121, *p* = 0.038; Fig. 6f) showed significant associations with DFS, where IP5 was associated with a favorable outcome and IP3 was associated with the worst DFS. Patients in the IP4 group (distinguished from those in the IP5 group by high PIL count) had the same poor DFS rate as those in the IP3 group. In contrast, among patients with the TNBC subtype, those in the merged IP1/2 group had significantly improved DFS than patients in the IP5 group (log-rank = 6.419, *p* = 0.040; Fig. 6e). Similar trends were observed for patients with the luminal B-HER2 and HER2+ subtypes, but the association with DFS was not significant for either subtype (Fig. 6c, d). Among patients with the luminal A subtype, only two were included in the IP1 and IP2 groups, and the DFS curves of the merged-IP groups overlapped without a significant association in terms of DFS (Fig. 6a). Both univariable and multivariable analyses showed that the IP had no significant associations with DFS (Supplementary Table 2).

### Spatial differences in the immune cell subpopulations

**Fig. 7:**
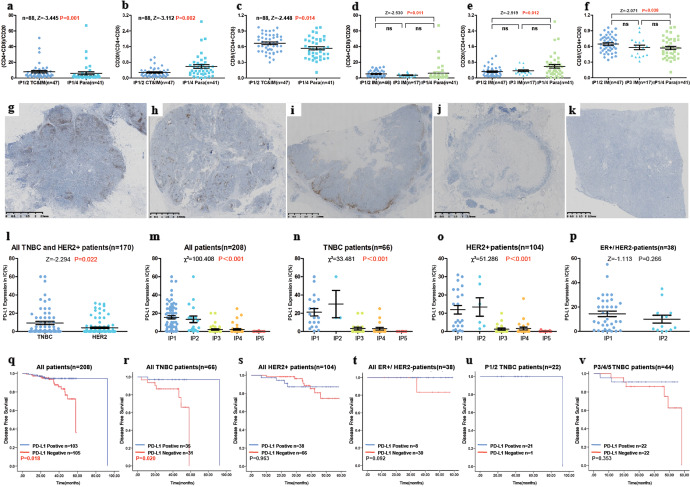
Different immune cell subpopulations in tumor-associated areas and para-tumor areas for all five IPs identified in this study. The ratio of T lymphocytes (CD4− and CD8-positive) to B lymph cells (CD20-positive) **a**, B lymph cells (CD20-positive) to T lymph cells (CD4− and CD8-positive) **b**, and cytotoxic T cells (CD8-positive) to T lymph cells **c** among IP1/2 TCs and IMs and IP1/4 Paras. The ratio of T lymph cells to B lymph cells **d**, B lymph cells to T lymph cells **e**, and cytotoxic T cells (CD8-positive) **f** to T lymph cells among IP1/2 IMs, IP3 IMs, and IP1/4 Paras. PD-L1-positive immune cells (SP142, brown DAB staining) observed with all five patterns **g**–**k**. PD-L1 expression in immune cells among all five patterns for all patients **m**, all patients with TNBC or HER2+ tumors **l**, all patients with TNBC **n**, all HER2+ patients **o**, and all luminal HER2− patients **p**. Kaplan–Meier curves for disease-free survival according to the median expression level of PD-L1 in all patients **q**, all patients with TNBC **r**, all HER2+ patients **s**, all ER+/HER2− patients **t**, all patients with TNBC in the IP1 and IP2 groups **u**, and all patients with TNBC in the IP3, IP4, and IP5 TNBC groups **v**. IP Immunoarchitectural pattern, TC tumor center, IM invasive margin, Para para-tumor area, TNBC triple-negative breast cancer.

We selected 77 typical cases of different IPs (IP1 = 28, IP2 = 19, IP3 = 17, and IP4 = 13) to compare the subpopulations of TILs located in stromal tumor center (TC) invasive margin (IM), and PILs in Para. The TIL subpopulations in the TCs and IMs of IP1/2 group were averaged to obtain total TIL-subpopulation densities to represent “hot tumors” group. Likewise, we combined each PIL subpopulation (CD4, CD8, and CD20) of IP1/4 group to represent “hot Para” group. We compared the ratio of T cells (CD4+ CD8+) to B cells (CD20+), and the ratio of cytotoxic T cells (CD8+) to T cells in these groups. We found that the proportion of T cells (z = −3.455, *p* = 0.001) and cytotoxic T cells (z = −2.448, *p* = 0.014) in “hot tumor” group was significantly higher than that in “hot Para” group. In contrast, the proportion of B cells was significantly higher in “hot Para” group than that in “hot tumor” group (z = −3.112, *p* = 0.002; Fig. 7a–c).

We then compared the ratio of T cells to B cells, and the ratio of cytotoxic T cells to T cells for the following two pairs: IM of IP3 and IP1/2; IM of IP3 and Para of IP1/4. No significant differences were found (Fig. 7d–f).

### The PD-L1-positive rate differed significantly between IPs and correlated with DFS in all cases and TNBC subtypes

From the 579 IBC-NSTs cases, 208 typical cases were selected for PD-L1 staining and were divided into two cohorts. Cohort 1 contained all enrolled patients with TNBC and HER2+ cases (with all IPs). Cohort 2 contained all ER+/HER2− patients with IP1 and IP2 was used as a control to eliminate the effect of molecular subtype on PD-L1 expression in lymphocyte predominant breast cancer (LPBC) patients. PD-L1 was mainly expressed in immune cells (ICs) (103/208, 49.52%), including lymphocytes, macrophages, dendritic cells, plasma cells, and granulocytes, but was rarely expressed in tumor cells (13/208, 6.25%). Positive ICs were characterized by dark brown punctate or linear membrane staining. The spatial distributions of positive ICs resembled those of the IPs (Fig. 7g–k).

PD-L1-expression levels were significantly higher in the TNBC subtype than in the HER2+ subtype (*p* = 0.0031, *n* = 170; Fig. 7l). PD-L1 positivity was significantly associated with IPs (*p* < 0.001, *n* = 208; Fig. 7m), where ICs in both the IP1 (84.06%) and IP2 (89.47%) groups showed the highest positive rates, followed by IP3 (31.71%) and IP4 (31.82%), while ICs in the IP5 group had the lowest expression rate (2.86%) (Supplementary Table 3). The same tendency was also observed in the TNBC (*p* < 0.001, *n* = 66) and HER2+ subtypes (*p* < 0.001, *n* = 104; Fig. 7n, o). In cohort 2, PD-L1 expression in ER+/HER2− patients was high in both IP1 and IP2 groups, with no significant difference (*p* = 0.31, *n* = 38; Fig. 7p).

Survival analysis revealed that in all 208 cases, including 66 TNBC cases, a PD-L1-positive status was significantly associated with a better prognosis (*p* = 0.018 and *p* = 0.020, respectively; Fig. 7q, r). No predictive significance was found for survival in patients with the HER2+ and ER+/HER2− subtypes (Fig. 7s, t). To eliminate the influence of TIL count on DFS in TNBC, we subdivided the TNBC cases into those with high-TIL count (P1/2) and low-TIL count (P3/4/5). No significant predictive value was found for either group, based on PD-L1 expression (Fig. 7u, v).

### Tumor-mutation signature and immune-response gene expression

We selected 40 cases from the five IPs to detect TMBs based on whole-exome sequencing. No significant differences were observed (Supplementary Figure 4). We then compared the differences between oncodriver genes using the Catalog of Somatic Mutations in Cancer database (http://cancer.sanger.ac.uk/census) and found that 44% (141/320) of the genes had a lower mutation rate in the IP5 group than in the other IP groups, including breast-cancer-specific oncodriver genes (*TP53*, *ERBB2*, *MAP3K4*, *BRCA1*, *ERBB4*, and *PIK3CA*).

By comparing the expression of immune-response genes between TCs and IMs in the IP1/2 (*n* = 6) and IP1/4 Para (*n* = 8) groups, we identified a cluster of highly expressed genes in IP1/2 TCs and IMs (Supplementary Fig. 4), including T cell activation-associated genes (*CD8A*, *CXCR6*, *IL-12A*, *IL-8*, and *EBI3*), the immune checkpoint gene *ICOS* (also known as *CTLA4*), and MHC II molecules, such as *HLA-DRB1*. Equivalently, high expression of genes related to B-cell activation—including *CD40LG* and *CD79B*—MHC molecules, *CD1D*, and B/T/Th1 cell activation genes— *CCL21*, *IL12B*, and *PTPRC*—was observed in IP1/4 Para (Supplementary Fig. 4).

## Discussion

In this study, we used a traditional software-assisted assessment system for assessing IPs of IBC-NSTs on whole slide images (WSIs). This rigorous assessment system enabled us to establish the classification criteria for IPs by calculating the areas of the lymphatic nuclei instead of those of whole cells and accurately distinguished IM and TC regions.

At the morphological level, the densities and spatial distributions of TILs are heterogenous in different areas (TC, IM, and Para) of the same block, but FFPE blocks of the same tumor are homogenous, as reported by Mani^10^. In our study, specifically, TIL count in TCs was similar to those in IMs; high-TC TIL count indicated high-IM TIL count, but the reverse was not necessarily true. No cases with high-TC TIL density and low IM density were found. Tumors with high-TIL densities in both areas fulfill the LPBC criteria; thus, TCs provide sensitive and corroborative evidence to confirm the LPBC status, thereby distinguishing IP1/2 from other IPs. In IP1 and IP2, the TIL density and subpopulation in TC and IM showed high accordance with each other. This homogeneity allowed us to propose a hypothesis that TILs in TCs disperse from IMs, which are regions characterized by higher vessel density^11^. We then demonstrated that patients with IP1/2 had the best DFS in TNBC and better DFS than that for IP3 (high TIL density in IMs only) in luminal B-type cases, further suggesting that high-TIL and CD8+ T-cell densities reflect anti-tumor immunity and are indicative of a good prognosis^12,13^. IP1 and IP2 are demarcated based on higher PIL levels in IP1 compared with those in IP2.

The finding that the density of TILs was higher in IMs than in TCs was also reported by Mi et al.^14^, suggesting the distinct role of IMs in the tumor immune architecture. They further discovered that the IM is multifaceted and may serve pro- and anti-tumor functions simultaneously with higher CD8+ expression and more FOXP3+ cells. We deduced that those cases with higher CD8+ TILs in IMs might show the same pattern as IP1/2 group, whereas the rest cases belonged to IP3. In IP3, TILs were mainly restricted to the IM region and maintained at a medium level (>10%), and their subcellular populations were also altered, with a trend toward reduced CD8/(CD4+ 8) ratios and increased CD20/(CD4+ 8) ratios, though the alterations were not significant. A similar pattern of “altered immune tumors” was reported for colorectal cancer^15^, based on the expression levels and spatial distributions of CD3 and CD8. The density and subpopulation heterogeneity of TILs in IP1/2 and IP3 indicate their multifaceted functions, which was supported by their differential DFS in luminal B-type and TNBC cases.

Histological grade influence on IP predictive ability in the luminal B subtype. In this study, all five individual IPs of luminal B and merged IPs of TNBC cases showed significant associations with DFS. Since previous reports confirmed that histological grade is a significant independent factor for DFS in luminal but not TNBC patients^16^, it is reasonable to verify whether the predictive ability of IPs is attributed to the histological grade. Using the chi-square test, we found that the IP distribution in the luminal B subtype differs significantly in three histological grades. The DFS differences by the Kaplan–Meier survival were no longer evident when stratified by grade. These findings indicated that the DFS predictive ability of IPs in the luminal B subtype may have a close association with histological grade. While in the TNBC cohort, the chi-square test failed due to the existence of many variables smaller than 5, which confirmed the deduction that the skewed distribution of TNBC in histological grades could be a major factor contributing to its low predictive ability^16^.

Most studies have focused on the immune microenvironment in the tumor area. In this study, we expanded the scope of lymphocyte evaluation to the Para. As expected, corresponding to “hot” immune tumors, we found another equally obvious “hot” immune region localized in the IP1 and IP4 Para with high frequency of lobular involvement. This distinction could be used to stratify LPBC into IP1 and IP2, and “cold” immune tumors into IP4 and IP5. The fact that no significant difference in the frequency of lobular involvement occurred between IP1 and IP4 indicates that the immune status within a tumor area is not responsible for driving lobular involvement.

The subpopulation of IC and gene mutations in different IPs. Previously, it was discovered that infiltrating ICs not only function to control tumor growth and progression, but also help to create an immunosuppressive environment in which the tumor can thrive^17^. IC subpopulations in the tumor area, combined with density and location, could predict the survival of patients with colorectal cancer more accurately than the classical tumor–node–metastasis (TNM) system^18^, where a high CD8/CD3-density ratio correlated with a good prognosis^12^. However, ICs in IM are multifaceted^19^, and may exhibit pro- and anti-tumor functions simultaneously based on the different levels of CD8 and FoxP3^14^. Furthermore, B cells and plasma cells can also adopt either effector or regulatory phenotypes, and hence, exhibit positive or negative anti-tumor associations depending on the contextual factors^19–21^. In this study, statistical analysis showed that T cells and CD8-positive cytotoxic T cells were significantly more abundant in tumor areas of IP1 and IP2 than in Paras, further suggesting that T cells (especially cytotoxic T cells) exhibit anti-tumor functions in tumor areas. In contrast, high density of B cells in the Para might promote tumor progression, considering the para-tumor immune microenvironment mentioned above. Genome-wide mRNA-expression analysis in ICs revealed a cluster of immune-related genes that were differentially expressed in the tumor area and para-tumor area, which deserves further exploration in terms of the function and clinical significance. The median CD20/(CD4+ CD8) and CD8/(CD4+ CD8) ratios in IP3 IMs were between those of IP1/2 IMs and IP1/4 Paras, which might indicate a compromised status between the host and tumor.

IP5 was characterized by very low or no IC infiltration in both the TC and Para, with similar weak immune reactions or immune ignorance immune type described by Camus and colleagues^12^. Patients in IP5 mainly presented a lower histological grade (88%) and a luminal molecular type (86%). We speculate that the determining factor for different immune reactions within all five IPs was the tumor antigen load. However, patients in IP1 and IP2 did not show a higher TMB than IP5. We identified 320 oncodriver gene mutations in 40 patients. Among these, nearly half (44%) exhibited a markedly lower mutation rate in IP5, including *TP53*, *ERBB2/4*, *MAP3K4*, *BRCA1*, and *PIK3CA*. Further cases are needed to verify whether the heterogeneous immunogenicity of IBC is attributable to driver gene mutation signatures, as suggested in prior studies^22^.

IPs can be used in preliminary screening for PD-L1 expression. PD-L1 expression in the five IPs showed very similar trends in the following three groups: all selected cases, the TNBC subtype, and the HER2+ subtype. These highly repetitive expression trends suggest that PD-L1 expression coincides well with the IP-classification scheme. Therefore, we speculate that PD-L1 expression was closely related to the density of TILs, a phenomenon reported by other researchers^23–25^, rather than the tumor molecular subtypes. Through survival analysis, we found that PD-L1 expression was significantly associated with a better DFS for all 208 cases and in patients with TNBC. Surprisingly, the HER2+ subtype cases, with a similar trend in PD-L1 positivity and different IPs in TNBC, did not show the same predictive value in terms of PD-L1 expression. Considering that the PD-L1 expression level in TNBC is significantly higher than that in the HER2+ subtype, we speculate that the PD-L1-expression level is a better predictor of DFS than the PD-L1-positivity status (1% cut-off). Therefore, it is clinically more meaningful to provide exact PD-L1-positive IC percentages than to simply provide a positivity status^26^. Moreover, owing to the close relationship and similar DFS predictive value between PD-L1 expression and IPs, TNBC cases that intersected with IP1/2 were mostly PD-L1 positive, whereas cases with complete TIL deficiency in IP5 could be considered PD-L1 negative. Therefore, predicting the PD-L1-expression status using IP classification based on H&E staining is feasible and can be considered for preliminary screening of patients with IBC-NST. It is recommended to report both TILs and PD-L1 as a combined immune-oncological biomarker in daily practice^27^.

According to the conception of tumor immunoediting, IP of IBC represents the final immune manifestation of ductal carcinoma in situ (DCIS) progression. By comparing of pure DCIS, DCIS mixed synchronous IBC, and IBC, the immune cell subsets and spatial distribution were found highly variable^28,29^. Further studies need to focus on certain key immunomodulatory switches, such as CD103^30^ and CTLA4, in specific IP during the progression from DCIS to IBC, to trace the formation of the IPs and select the more progressive DCIS for early immunotherapy.

In summary, we established five morphological IPs by studying the immune architecture of IBC-NSTs in terms of density, spatial distribution, subpopulation of lymphocytes, TMB, and immune-associated gene expression profiles. The clinical significance of the different IPs is as follows: (1) high-TIL counts in the TC represented the most robust indicator for LPBC, with a high proportion of CD8+ T cells, indicating favorable DFS in TNBC; (2) high PIL counts with a high proportion of CD20+ B cells indicated poor DFS similar to TILs mainly in the IM in luminal B subtype IBC; (3) PD-L1 positivity significantly correlated with the IC counts and could be predicted by IPs; and (4) IPs of IBC-NST might be a potential prognosis factor, especially in the TNBC and luminal B subtypes.

There are several limitations to our investigation. First, this study was a retrospective evaluation, so it is not clear whether the IP system may help predict the therapeutic responses. Second, the short clinical follow-up period for these patients further restricted the predictive value in terms of survival, especially for luminal tumors, where late recurrences are common. Third, future studies should explore the genetic variation underling tumor IPs to screen for novel markers that can serve as potential immunotherapeutic targets in breast cancer. Despite these limitations, our study provides unique insights into the tumor microenvironment architecture and its potential clinical prospects.

## Methods

### Cohort enrollment

The study cohort consisted of 579 consecutively archived IBC-NST samples that were surgically excised at the time of diagnosis between 08/2015 and 08/2018. All enrolled patients provided written informed consent to use these samples for translational research, as approved by the Ethics Commission of the General Hospital of China PLA (approval number ky-2020-1-4) and the study was compliant with the ‘Guidance of the Ministry of Science and Technology (MOST) for the review and Approval of human Genetic Resources’, China. For TIL quantification, all specimens were reviewed for pathomorphology on H&E-stained sections to select the most representative tumor block. Biomarker expression levels and other clinicopathological features were extracted from the archives. Histological grades were scored according to the Nottingham semi-qualitative scoring system^31^. Tumor molecular subtyping (expression of the associated parameters, including ER, PR, HER2, and Ki-67) and TNM staging were evaluated based on World Health Organization’s classification of breast tumors (5th edition)^31^. All H&E-stained and IHC-stained sections were scanned using a KFBIO Scans cope high-resolution scanner at 40× magnification (Konfoong Biotech, Ningbo, China), and all parameters assessed in this study were examined using WSIs.

### Evaluating key TIL parameters and determining IPs

TILs are defined as mononucleated lymphoid cells, which include lymphocytes and plasma cells. The overall assessment of stromal TILs (sTILs), rather than hotspots of intense infiltration, was conducted using H&E-stained sections to eliminate interior heterogeneity. To elucidate the spatial distribution of TILs, the tumor areas were divided into the TC and IM as per the guidelines of the International Immuno-Oncology Biomarker Working Group^19,32^ (Fig. 1). We used a traditional software (StrataQuest 6.0.213, TissueGnostics, Vienna, Austria)-assisted assessment system^33^ to precisely evaluate the ratio of TILs or PILs to the stromal area in TCs, IMs, and Para. Lobular involvement defined as invasive tumor cells expand into the lobules in Para, which was commonly seen in BC.

The procedural steps are outlined in Fig. 1. Five consecutive complementary steps of software-assisted manual assessment were performed: 1. The boundaries of tumor area (red) and Para (yellow) were manually delineated by pathologists. Then the confounder regions (gray) that were to be excluded from these areas were annotated, including necrosis, tertiary lymphoid structures, intermixed normal tissue; 2. The ratio of stromal area to tumor area or Para was manually assessed by two pathologists to eliminate bias from the variability of stromal to tumor area ratio between different cases. 3. The IM area was automatically mapped by the software, and thus, IM and TC were segmented; 4. The numbers of TILs (red) and PILs (red) in TC, IM, and Para were automatically labeled and counted. 5. The ratio of TILs or PILs nucleus area to the stromal area in TC, IM, and Para was calculated.

By calculating the ratio of nucleus to the whole lymphocyte area, a threshold value of 20% ratio of the lymphatic nucleus area to the stromal area in the TC was used as a cut-off for the quantity scores of consecutive sTILs, in order to identify LPBC (50% stromal TILs) samples. A cut-off ratio of 10% lymphatic nucleus area to stromal area in the IM was used to distinguish samples with intermediate and low immunoscores. We further included PILs, based on a cut-off ratio of 20% lymphatic nucleus area to stromal area to investigate the influence of tumor immunity on the Para. Finally, five different types of IPs were determined, as shown in Fig. 2.

### IHC

FFPE tissue specimens were sectioned at 4 μm thickness. Antibodies against CD4 (clone number UMAB64, ZSbio, Wuxi, China), CD8 (clone number SP16, ZSbio), CD20 (clone number L26, ZSbio), and PD-L1 (clone number SP142, Ventana Medical Systems, Oro Valley, AZ) were used for IHC, which was performed using a Benchmark Ultra System, the OptiView DAB IHC Detection Kit (Ventana Medical Systems). A rabbit monoclonal negative immunoglobulin (Ventana Medical Systems) was used as negative control, and FFPE tonsil tissue stained with PD-L1 was used as positive control. All negative and positive controls were used for each batch of samples. The PD-L1 staining scores of TILs (cut-off value: 1% staining of any intensity) were evaluated by two board-certified pathologists according to the IM passion 130 Trial criteria^25,34^. The positivities of CD markers were assessed based on a digital-pathology computational workflow (StrataQuest 6.0.213 software for WSIs^14,33^. The densities of CD4-, CD8-, and CD20-positive lymphocytes were evaluated through counting the numbers of nuclei encompassed by DAB-stained cytoplasm and membrane (Supplementary Fig. 1). A threshold was established to ensure the accurate labeling of positive cells for each region. The output parameters are presented as the number of positive lymphocytes/mm^2^ (for TCs, IMs, and Para).

### DNA isolation and whole-exome sequencing

We selected samples from 40 patients with different molecular subtypes and matched FFPE and blood samples to detect germline mutations. Genomic DNA was extracted using a QIAamp DNA FFPE Tissue Kit or a DNeasy Blood & Tissue Kit (Qiagen, Hilden, Germany). Whole-exome sequencing libraries were constructed using an NEBNext Ultra DNA Library Prep Kit (Illumina, San Diego, CA) and according to the manufacturer’s protocol. The average whole-exome sequencing depth was 100× for FFPE tumors samples, and 60× for their normal tissue counterparts.

### RNA isolation and Illumina immune-response gene panel sequencing

Macrodissections were performed on whole H&E-stained sections to collect TILs from tumor areas and PILs from Para. Total RNA was extracted using a miRNeasy FFPE Kit (Qiagen, Hilden, Germany) following the manufacturer’s instructions. RNA-sequencing libraries were constructed using the AmpliSeq Immune Response Panel (Illumina, San Diego, CA) according to the manufacturer’s protocol. Then, the quality of the library (2 × 150 base-pair, paired-end reads) was checked, and it was sequenced using a HiSeq 2500 System (Illumina, San Diego, CA).

### Statistical analysis

Tumor sizes, metastatic lymph nodes, and the scores for CD4, CD8, CD20, PD-L1, ER, PR, and Ki-67 staining were treated as continuous variables. They were tested for normality and homogeneity of variance and were found not normally distributed and not homogenous. Non-parametric tests (Mann-Whitney U test of Two-Independent Samples; Kruskal-Wallis H test of K Independent samples) were conducted to assess the difference between groups. Some of these parameters were missing, and therefore, omitted in subsequent analysis. We assessed the correlations between patterns and categorical variables (clinical stage, histological grade, HER2 status, molecular type, lobular involvement, and cancerous embolus) by performing χ² tests for trends or Fisher’s exact tests. Survival endpoints were evaluated as DFS. Survival curves were prepared based on Kaplan–Meier estimates and compared using the log-rank test. The Cox proportional hazards model was used to perform univariable and multivariable analyses to further identify the variables independently associated with DFS. Statistical analyses were performed using SPSS 23 (IBM Corporation, Somers, NY). All statistical tests were 2-sided, and *P* values < 0.05 were considered as statistically significant.

### Reporting summary

Further information on research design is available in the Nature Research Reporting Summary linked to this article.

## Supplementary information

Supplementary Data

Reporting Summary Checklist

Supplementary Figures Caption

## Data Availability

DNA-seq and RNA-seq datasets were uploaded on National center for biotechnology information (NCBI) under the Accession Number of PRJNA786713. The datasets generated and/or analyzed during the current study are available from the corresponding author on reasonable request.
