# Supplementary material for: Immunoarchitectural patterns as potential prognostic factors for invasive ductal breast cancer
Source: NPJ Breast Cancer. 2022 Feb 28;8:26. doi: 10.1038/s41523-022-00389-y (PMC8885796; doi:10.1038/s41523-022-00389-y)
Supplement: Supplementary file 1 — Supplementary Data [file 41523_2022_389_MOESM1_ESM.pdf]

Supplementary Figure 1

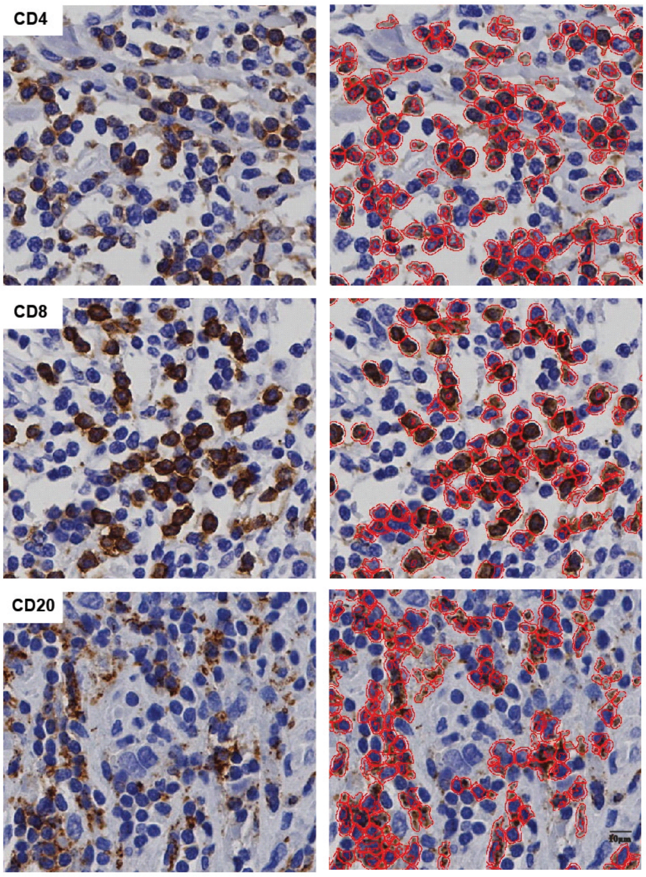

Supplementary Figure 2

Lobular involvement

		IP 1	IP 2	IP 3	IP 4
IP2	Pearson	10.768			
	P-value	0.001			
IP3	Pearson	11.748	1.629		
	P-value	0.001	0.202		
IP4	Pearson	21.355	56.602	73.968	
	P-value	3.82E-06	6.79E-13	7.94E-18	
IP5	Pearson	18.46	1.213	0.246	102.306
	P-value	1.74E-05	0.271	0.62	4.76E-24

Cancerous embolus

		IP 1	IP 2	IP 3	IP 4
IP2	Chi square	0.471			
	P-value	0.493			
IP3	Chi square	2.569	0		
	P-value	0.109	1		
IP4	Chi square	10.28	0.984	3.92	
	P-value	0.001	0.321	0.048	
IP5	Chi square	1.934	0.062	0.233	8.825
	P-value	0.164	0.803	0.629	0.003

HER2 status

		IP 1	IP 2	IP 3	IP 4
IP2	Pearson	0.009			
	P-value	0.924			
IP3	Pearson	2.53	1.278		
	P-value	0.112	0.407		
IP4	Pearson	2.301	1.123	0.22	
	P-value	0.129	0.445	0.883	
IP5	Pearson	14.635	6.278	4.497	5.801
	P-value	1.31E-04	0.031	0.034	0.016

Tumor grade

		IP 1	IP 2	IP 3	IP 4
IP2	Mann-Whitney	592			
	Z-value	-0.76			
	P-value	0.447			
IP3	Mann-Whitney	2568	801		
	Z-value	-4.122	-1.863		
	P-value	3.75E-05	0.063		
IP4	Mann-Whitney	2616.5	830	6504	
	Z-value	-5.542	-2.740	-1.427	
	P-value	3.00E-08	0.006	0.154	
IP5	Mann-Whitney	3830	1252.5	10489	13808
	Z-value	-8.48	-4.369	-4.486	-3.109
	P-value	2.24E-17	1.25E-05	7.25E-06	0.002

Clinical stage

		IP 1	IP 2	IP 3	IP 4
IP2	Mann-Whitney	640			
	Z-value	-0.5			
	P-value	0.617			
IP3	Mann-Whitney	3180.5	900.5		
	Z-value	-3.142	-1.474		
	P-value	0.002	0.14		
IP4	Mann-Whitney	4027.5	1138	6620.5	
	Z-value	-2.211	-0.928	-1.422	
	P-value	0.027	0.364	0.155	
IP5	Mann-Whitney	7460.5	2110.5	13380	15480.5
	Z-value	-2.903	-1.287	-0.622	-1.03
	P-value	0.004	0.198	0.534	0.303

ER

		IP 1	IP 2	IP 3	IP 4
IP2	Mann-Whitney	455			
	z-value	-2.157			
	P-value	0.031			
IP3	Mann-Whitney	2571.5	1028		
	Z-value	-3.74	-0.116		
	P-value	1.84E-04	0.908		
IP4	Mann-Whitney	2741	1177	6754.5	
	Z-value	-4.63	-0.338	-0.754	
	P-value	4.00E-07	0.735	0.451	
IP5	Mann-Whitney	3181.5	1667	9255.5	11698.5
	Z-value	-8.252931	-2.276	-5.133	-4.681
	P-value	1.55E-16	0.023	2.85E-07	3.00E-07

Tumor size

		IP 1	IP 2	IP 3	IP 4
IP2	Mann-Whitney	461			
	z-value	-1.979			
	P-value	0.048	1		
IP3	Mann-Whitney	3638.5	797.5		
	Z-value	-0.466	-1.654		
	P-value	0.641	0.098	1	
IP4	Mann-Whitney	3761	717	5777	
	Z-value	-1.802	-2.926	-2.486	
	P-value	0.072	0.003	0.013	1
IP5	Mann-Whitney	8509	1721.5	12992	13962
	Z-value	-0.172	-2.006	-0.837	-2.149
	P-value	0.864	0.045	0.403	0.032

Node No.

		IP 1	IP 2	IP 3	IP 4
IP2	Mann-Whitney	627.5			
	z-value	-0.363			
	P-value	0.717			
IP3	Mann-Whitney	3316.5	872		
	Z-value	-1.66	-1.329		
	P-value	0.097	0.184		
IP4	Mann-Whitney	4129.5	1086.5	6829	
	Z-value	-1.101	-1.007	-0.691	
	P-value	0.271	0.314	0.489	
IP5	Mann-Whitney	8006.5	2097	13037.5	16191
	Z-value	-1.142	-1.039	-0.978	-0.143
	P-value	0.253	0.298	0.328	0.886

PR

		IP 1	IP 2	IP 3	IP 4
IP2	Mann-Whitney	533			
	z-value	-1.343			
	P-value	0.18			
IP3	Mann-Whitney	2766.5	967		
	Z-value	-3.176	-0.532		
	P-value	1.49E-03	0.595		
IP4	Mann-Whitney	3282	1170.5	7003.5	
	Z-value	-3.223	-0.376	-0.279	
	P-value	1.27E-03	0.707	0.78	
IP5	Mann-Whitney	4255	1706.5	10816	12201
	Z-value	-6.543	-2.087	-3.31	-4.082
	P-value	6.032E-11	0.037	9.33E-04	4.50E-05

Molecular type

		IP 1	IP 2	IP 3	IP 4
IP2	Mann-Whitney	534.5			
	z-value	724.5			
	P-value	0.198			
IP3	Mann-Whitney	2679	900		
	Z-value	-3.475	-1.02		
	P-value	0.001	0.308		
IP4	Mann-Whitney	2927	994	6778	
	Z-value	-4.227	-1.449	-0.733	
	P-value	2.37E-05	0.147	0.463	
IP5	Mann-Whitney	3295	1216.5	9356	11906
	Z-value	-8.482	-3.9	-5.278	-4.679
	P-value	2.21E-17	9.63E-05	1.30E-07	2.89E-06

KI-67

		IP 1	IP 2	IP 3	IP 4
IP2	Mann-Whitney	588.5			
	z-value	-0.683			
	P-value	0.494			
IP3	Mann-Whitney	2097	653.5		
	Z-value	-5.054	-2.616		
	P-value	4.323E-07	8.89E-03		
IP4	Mann-Whitney	2119	674.5	6652	
	Z-value	-6.148	-3.208	-0.935	
	P-value	7.871E-10	1.34E-03	0.35	
IP5	Mann-Whitney	2278.5	770.5	9446	12153.5
	Z-value	-9.435	-4.961	-4.815	-4.117
	P-value	3.904E-21	7.01E-07	1.00E-06	3.80E-05

Supplementary Figure 3

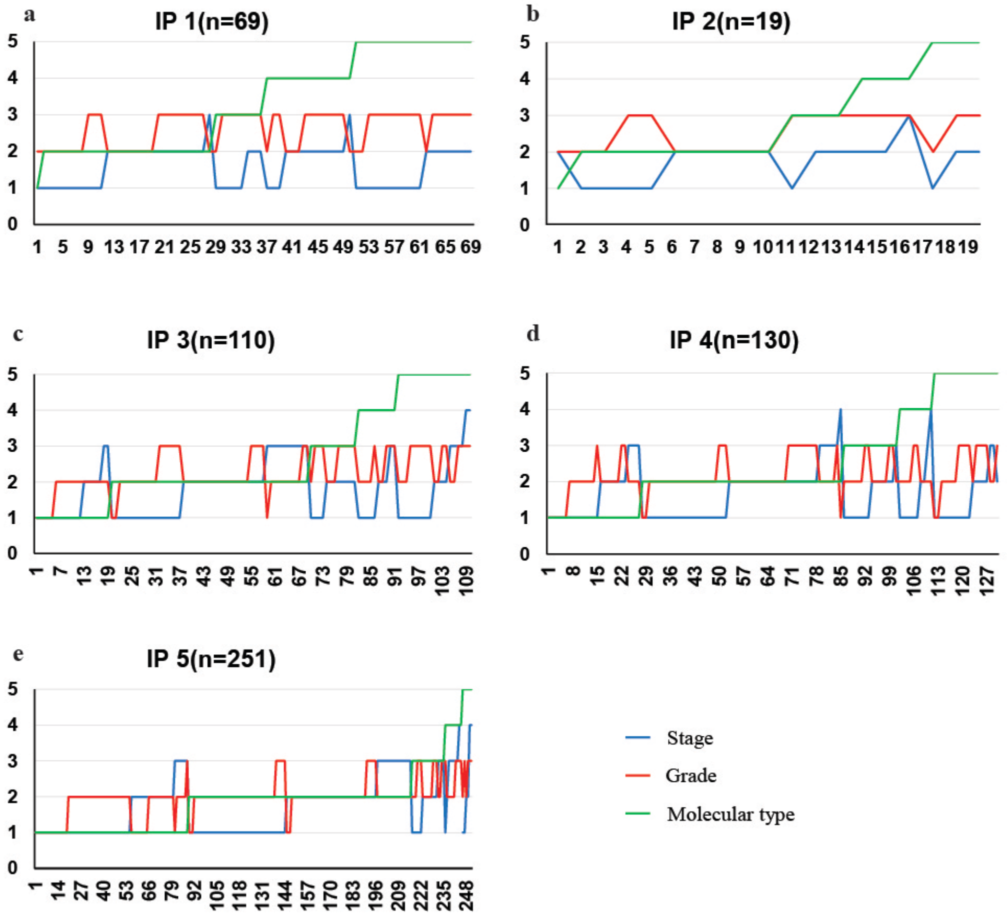

Supplementary Figure 4

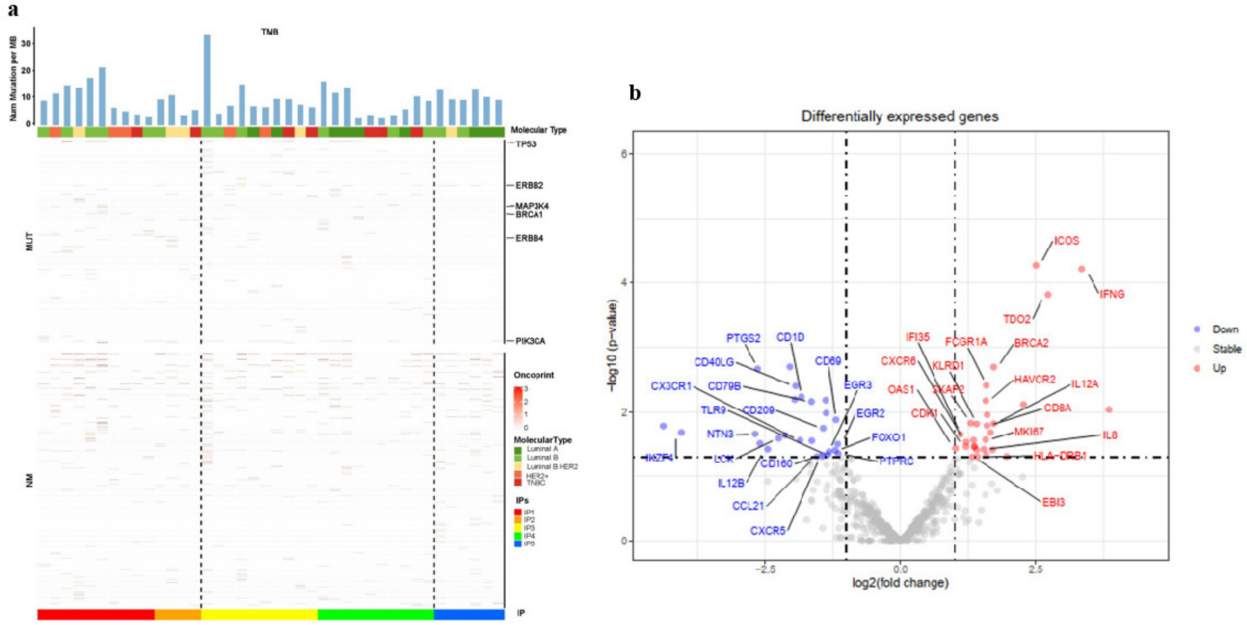

Supplementary Table 1. The clinicopathological significance of 5 immunoarchitectural patterns.

	IP1	IP2	P3	IP4	IP5	Sum
Lobular involvement	Chi-square Tests	n=579	Pearson χ2=120.68	P=3.83E-25		
Yes	47 (68.12%)	5 (26.32%)	46 (41.82%)	121 (93.08%)	98 (39.04%)	317
No	22 (31.88%)	14(73.68%)	64 (58.18%)	9 (6.92%)	153 (60.96%)	262
Cancerous embolus	Chi-square Tests	n=579	Pearson χ2=14.22	p=0.007		
Yes	8 (11.59%)	4 (21.05%)	23 (20.91%)	42 (32.31%)	47 (18.73%)	124
No	61 (88.41%)	15(78.95%)	87 (79.09%)	88 (67.69%)	204 (81.27%)	455
Histological grade	Kruskal-Wallis Tests	n=579	χ2=84.84	P=1.64E-17		
Grade 1	0 (0.00%)	0 (0.00%)	8 (7.27%)	11 (8.46%)	36 (14.34%)	55
Grade 2	26 (37.68%)	9 (47.37%)	66 (60.00%)	88 (67.69%)	185 (73.71%)	374
Grade 3	43 (62.32%)	10(52.63%)	36 (32.73%)	31 (23.85%)	30 (11.95%)	150
Clinical stage	Kruskal-Wallis Tests	n=577	χ2=12.06	P=0.017		
I +II	67 (79.10%)	18(94.74%)	89 (80.91%)	113 (87.60%)	209 (83.60%)	496
III+IV	2 (2.90%)	1 (5,26%)	21 (19.09%)	16 (12.40%)	41 (16.40%)	81
Tumor size	Kruskal-Wallis H Tests	n=577	χ2=56.323	P=1.7159E-11		
Tumor size(cm)	1.95±0.09	2.76±0.35	2.25±0.16	1.72±0.07	2.15±0.11	
Metastatic lymph node	Kruskal-Wallis H Tests	n=579	χ2=3.994	P=0.407		
Node number	0.71±0.25	0.63±0.30	1.99±0.50	2.07±0.54	1.10±0.15	
Molecular type	Chi-square Tests	n=579	Pearson χ2==96.41	P=1.63E-13		
Luminal A	1 (1.45%)	1 (5.26%)	19 (17.27%)	27 (20.77%)	88 (35.06%)	136
Luminal B	27 (39.13%)	9 (47.37%)	50 (45.45%)	58 (44.62%)	128 (51.00%)	272
Luminal HER2	8 (11.59%)	3 (15.79%)	12 (10.91%)	16 (12.31%)	19 (7.57%)	58
HER2+	14 (20.29%)	3 (15.79%)	10 (9.09%)	10 (7.69%)	10 (3.98%)	47
TNBC	19 (27.54%)	3 (15.79%)	19 (17.27%)	19 (14.62%)	6 (2.39%)	66
Sum	69 (100%)	19 (100%)	110 (100%)	130 (100%)	251 (100%)	
Postive rate of markers	Kruskal-Wallis H Tests					
ER	30.19±4.46	53.95±9.05	53.85±3.58	58.55±3.13	76.39±1.40	
	n=579	χ2=82.633	P= 4.8191E-17			
PR	21.38±3.72	35.16±8.82	38.81±3.60	38.02±3.21	53.37±2.15	
	n=579	χ2= 50.657	P= 2.6322E-10			
Ki-67	46.16±2.59	42.63±4.74	29.90±1.97	26.82±1.65	18.86±0.81	
	n=579	χ2= 111.753	P= 3.0769E-23			
HER2 status	Chi-square Tests	n=579	Pearson χ2==17.53	P=0.002		
Postive	21 (30.43%)	6 (31.58%)	22 (20.00%)	27 (20.77%)	29 (11.55%)	105
Negative	48 (69.57%)	13(68.42%)	88 (80.00%)	103 (79.23%)	222 (88.45%)	474

Supplementary Table 2. Univariate and multivariate COX regression analysis of IPs and major clinicopathological characteristics for disease free survival in breast cancer patients.

	Univariable analysis				Multivariable analysis		
Complete cohort(n=579)	P-value	OR	95% CL	Complete cohort(n=579)	P-value	OR	95% CL
IPs	0.54	0.926	0.725-1.184	IPs	0.066	0.66	0.424-1.028
Cancerous embolus	0.024	2.232	1.11-4.488	Cancerous embolus	0.065	2.168	0.953-4.931
Lobular involvement	0.251	1.512	0.747-3.059	Lobular involvement	0.02	3.082	1.198-7.932
Tumor grade	0.13	1.645	0.864-3.131	Clinical stage	7.10E-07	3.189	2.016-5.043
Clinical stage	1.76E-08	3.571	2.293-5.56	Molecular type	1.50E-05	1.98	1.453-2.698
Molecular type	2.40E-05	1.648	1.307-2.078				
TNBC cohort(n=66)				TNBC cohort(n=66)			
IPs	0.032	2.145	1.067-4.311	Lobular involvement	0.113	288.021	0.262-316998.331
Cancerous embolus	0.034	5.075	1.132-22.758	Clinical stage	0.019	36.383	1.824-725.642
Lobular involvement	0.998	0.999	0.222-4.487				
Tumor grade	0.801	1.19	0.308-4.601				
Clinical stage	2.04E-04	5.725	2.28-14.374				
All HER2 cohort(n=105)				All HER2 cohort(n=105)			
IPs	0.64	1.103	0.73-1.667	Cancerous embolus	0.006	6.973	1.749-27.797
Cancerous embolus	0.03	3.553	1.134-11.134	Clinical stage	0	4.014	1.906-8.451
Lobular involvement	0.903	0.931	0.295-2.935				
Tumor grade	0.471	0.642	0.192-2.142				
Clinical stage	2.00E-04	3.679	1.852-7.308				
Luminal B cohort(n=272)				Luminal B cohort(n=272)			
IPs	0.127	0.738	0.5-1.09	Lobular involvement	0.066	6.93	0.878-54.715
Cancerous embolus	0.968	1.027	0.272-3.877				
Lobular involvement	0.047	8.067	1.032-63.033				
Tumor grade	0.739	0.78	0.18-3.377				
Clinical stage	0.123	1.892	0.842-4.253				
Luminal A cohort(n=136)				Luminal A cohort(n=136)			
IPs	0.666	11.9	0-921696.776	Ips	0.536	0.024	0-3327.651
Cancerous embolus	0.761	0.036	0-75948619.19	Cancerous embolus	0.89	0.107	0-5.99E+12
Lobules involvement	0.599	0.013	0-135616.755	Lobules involvement	0.678	0.034	0-299097.525
Tumor grade	0.547	0.007	0-77010.547	Clinical stage	0.607	0.064	0-2211.53
Clinical stage	0.645	0.048	0-18916.371	Tumor grade	0.544	0.01	0-28476.292

Supplementary Table 3. Description of statistical data of PD-L1 expression

	IP1	IP2	IP3	IP4	IP5
Cases	69	19	41	44	35
Positive cases	58	17	13	14	1
IC postive(%)	84.06	89.47	31.71	31.82	2.86
Median(%)	10	12	0.1	0.15	0
Mean(%)	15.5	13.31	1.74	2.25	0.07
95% Confidence interva（%）	11.98-19.3	5.88-20.74	0.72-3.65	0.68-3.83	0-0.13
