# Supplementary material for: Immunoarchitectural patterns as potential prognostic factors for invasive ductal breast cancer
Source: NPJ Breast Cancer. 2022 Feb 28;8:26. doi: 10.1038/s41523-022-00389-y (PMC8885796; doi:10.1038/s41523-022-00389-y)
Supplement: Supplementary file 2 — Reporting Summary Checklist [file 41523_2022_389_MOESM2_ESM.pdf]

nature portfolio

Corresponding author(s): Yanhong Tai, Shengqi Wang
Double-blind peer review submissions: write DBPR and your manuscript number here instead of author names.

Last updated by author(s): Dec 12, 2021

Reporting Summary

Nature Portfolio wishes to improve the reproducibility of the work that we publish. This form provides structure for consistency and transparency in reporting. For further information on Nature Portfolio policies, see our Editorial Policies and the Editorial Policy Checklist.

Statistics

For all statistical analyses, confirm that the following items are present in the figure legend, table legend, main text, or Methods section.

n/a	Confirmed	
☐	☒	The exact sample size (*n*) for each experimental group/condition, given as a discrete number and unit of measurement
☐	☒	A statement on whether measurements were taken from distinct samples or whether the same sample was measured repeatedly
☐	☒	The statistical test(s) used AND whether they are one- or two-sided<br>*Only common tests should be described solely by name; describe more complex techniques in the Methods section.*
☐	☒	A description of all covariates tested
☐	☒	A description of any assumptions or corrections, such as tests of normality and adjustment for multiple comparisons
☐	☒	A full description of the statistical parameters including central tendency (e.g. means) or other basic estimates (e.g. regression coefficient) AND variation (e.g. standard deviation) or associated estimates of uncertainty (e.g. confidence intervals)
☐	☒	For null hypothesis testing, the test statistic (e.g. *F*, *t*, *r*) with confidence intervals, effect sizes, degrees of freedom and *P* value noted<br>*Give P values as exact values whenever suitable.*
☒	☐	For Bayesian analysis, information on the choice of priors and Markov chain Monte Carlo settings
☐	☒	For hierarchical and complex designs, identification of the appropriate level for tests and full reporting of outcomes
☐	☒	Estimates of effect sizes (e.g. Cohen's *d*, Pearson's *r*), indicating how they were calculated

Our web collection on statistics for biologists contains articles on many of the points above.

Software and code

Policy information about availability of computer code

Data collection	*Provide a description of all commercial, open source and custom code used to collect the data in this study, specifying the version used OR state that no software was used.*
Data analysis	*Provide a description of all commercial, open source and custom code used to analyse the data in this study, specifying the version used OR state that no software was used.*

For manuscripts utilizing custom algorithms or software that are central to the research but not yet described in published literature, software must be made available to editors and reviewers. We strongly encourage code deposition in a community repository (e.g. GitHub). See the Nature Portfolio guidelines for submitting code & software for further information.

Data

Policy information about availability of data

All manuscripts must include a data availability statement. This statement should provide the following information, where applicable:

- Accession codes, unique identifiers, or web links for publicly available datasets
- A description of any restrictions on data availability
- For clinical datasets or third party data, please ensure that the statement adheres to our policy

The datasets generated and/or analyzed during the current study are available from the corresponding author on reasonable request.

Field-specific reporting

Please select the one below that is the best fit for your research. If you are not sure, read the appropriate sections before making your selection.

☒ Life sciences ☐ Behavioural & social sciences ☐ Ecological, evolutionary & environmental sciences

For a reference copy of the document with all sections, see nature.com/documents/nr-reporting-summary-flat.pdf

Life sciences study design

All studies must disclose on these points even when the disclosure is negative.

Sample size	This study was a retrospective evaluation. In multivariate Cox regression analysis, empirically the sample size should be 15 to 20 times the number of independent variables. In this study, Cox regression was used to evaluate the influence of various factors on patient survival outcomes. The number of influencing factors included in the regression model was 6, so the sample number was determined to be 120. In similar studies, the sample size was generally about 200. In this study we enrolled 579 cases, which could sufficiently ensure the representativeness and stability of the model estimation results.
Data exclusions	No data were excluded from the analyses.
Replication	To guarantee the measures reproducibility, two board-certified pathologists were employed in the course of measurements evaluation.
Randomization	All enrolled 579 cases were consecutively archived IBC-NST samples that were surgically excised at the time of diagnosis between 08/2015 and 08/2018, which guarantee the criterion of randomness.
Blinding	Blinding was not relevant to our study, because this study was a retrospective evaluation and did not group samples. Two pathologists, blind to each other and to the initial pathology report, performed the pathological evaluation.

Reporting for specific materials, systems and methods

We require information from authors about some types of materials, experimental systems and methods used in many studies. Here, indicate whether each material, system or method listed is relevant to your study. If you are not sure if a list item applies to your research, read the appropriate section before selecting a response.

Materials & experimental systems

n/a	Involved in the study	
☐	☒	Antibodies
☒	☐	Eukaryotic cell lines
☒	☐	Palaeontology and archaeology
☒	☐	Animals and other organisms
☒	☐	Human research participants
☒	☐	Clinical data
☒	☐	Dual use research of concern

Methods

n/a	Involved in the study	
☒	☐	ChIP-seq
☒	☐	Flow cytometry
☒	☐	MRI-based neuroimaging

Antibodies

Antibodies used	1, CD4: application IHC, ZSbio, Wuxi, China, catalog number ZM-0418, clone number UMAB64, lot number 19010408<br>2, CD8: application IHC, ZSbio, Wuxi, China, catalog number ZM-0508, clone number SP16, lot number 18112804<br>3, CD20: application IHC, ZSbio, Wuxi, China, catalog number ZM-0039, clone number L26, lot number 18110902<br>4, HRP,Hypersensitive enzyme-labeled HRP Goat Anti-Mouse/Rabbit IgG polymer: application IHC, ZSbio, Wuxi, China, catalog number PV-8000-1, lot number 19120115<br>5, PD-L1: application IHC, Ventana Medical Systems, Oro Valley, AZ, catalog number 07011571001,clone number SP142, lot number F16287<br>6, DAB, application IHC, Ventana Medical Systems, Oro Valley, AZ, catalog number 05269806001, lot number F12733
Validation	CD4, CD8, CD20 and SP142 are applied for the IHC assessment in human formalin-fixed FFPE tissues.
