# Supplementary material for: Immunoarchitectural patterns as potential prognostic factors for invasive ductal breast cancer
Source: NPJ Breast Cancer. 2022 Feb 28;8:26. doi: 10.1038/s41523-022-00389-y (PMC8885796; doi:10.1038/s41523-022-00389-y)
Supplement: Supplementary file 3 — Supplementary Figures Caption [file 41523_2022_389_MOESM3_ESM.docx]

**Supplementary Figure 1**. Objective cells for immunohistochemistry, identified using TissueGnostics Strata Quest 6 software.

**Supplementary Figure 2**. Statistical analysis for each clinical parameter studied.

**Supplementary Figure 3**. Major clinical characteristics of the five immunoarchitectural patterns.

**Supplementary Figure 4**. (a) Tumor mutational burden (TMB) and known oncodriver gene mutations in each of the five IPs identified in this study. (b) Expression of immune-response genes in the IP1/2 tumor areas and IP1/4 para-tumor areas. Genes upregulated in IP1/4 para-tumor areas are represented in the left panel, and genes upregulated in the IP1/2 tumor area are represented in the right panel.
